# Trefoil Factor Family Member 2: From a High-Fat-Induced Gene to a Potential Obesity Therapy Target

**DOI:** 10.3390/metabo11080536

**Published:** 2021-08-12

**Authors:** Abdelaziz Ghanemi, Mayumi Yoshioka, Jonny St-Amand

**Affiliations:** 1 Functional Genomics Laboratory, CREMI, Québec Genome Center, CHUL-CHU de Québec Research Center, Quebec, QC G1V 4G2, Canada; Abdelaziz.Ghanemi@crchudequebec.ulaval.ca (A.G.); mayumi.yoshioka@crchudequebec.ulaval.ca (M.Y.); 2 Department of Molecular Medicine, Faculty of Medicine, Laval University, Quebec, QC G1V 0A6, Canada

**Keywords:** trefoil factor family member 2, high-fat, metabolism, obesity

## Abstract

Obesity has its epidemiological patterns continuously increasing. With controlling both diet and exercise being the main approaches to manage the energy metabolism balance, a high-fat (HF) diet is of particular importance. Indeed, lipids have a low satiety potential but a high caloric density. Thus, focusing on pharmacologically targetable pathways remains an approach with promising therapeutic potential. Within this context, trefoil factor family member 2 (*Tff2*) has been characterized as specifically induced by HF diet rather than low-fat diet. TFF2 has also been linked to diverse neurological mechanisms and metabolic patterns suggesting its role in energy balance. The hypothesis is that TFF2 would be a HF diet-induced signal that regulates metabolism with a focus on lipids. Within this review, we put the spotlight on key findings highlighting this line of thought. Importantly, the hypothetical mechanisms pointed highlight TFF2 as an important contributor to obesity development via increasing lipids intestinal absorption and anabolism. Therefore, an outlook for future experimental activities and evaluation of the therapeutic potential of TFF2 inhibition is given. Indeed, its knockdown or downregulation would contribute to an antiobesity phenotype. We believe this work represents an addition to our understanding of the lipidic molecular implications in obesity, which will contribute to develop therapies aiming to manage the lipidic metabolic pathways including the absorption, storage and metabolism via targeting TFF2-related pathways. We briefly discuss important relevant concepts for both basic and clinical researchers.

## 1. Focusing on Lipids in Obesity and Metabolic Disorders

Since lipid metabolism is the main component of energy balance, we can expect that humans will have hormone(s) which are specific sensors for high fat (HF) intake in order to command the brain to stop eating. Still, no such hormone has been identified. The best strategy to develop a treatment for fat intake is to target the time before meal ingestion [1]. Based on this principle, our idea is to identify the HF-diet induced satiety hormone/peptide differentially regulated between 30 min to 3 h after HF compared to low fat/high carbohydrate (LF) meal and fasting in order to develop a potential drug given before the meal as a part of antiobesity therapy. Obesity, metabolic syndrome, cardiovascular disease (CVD) and type 2 diabetes (T2D) are complex multifactorial clinical conditions with altered energy balance and potentially similar candidate genes and therapeutic factors, involving complex gene–gene and gene–environment interactions [2,3,4,5,6,7,8,9,10,11,12,13,14,15,16,17,18]. Dietary lipids are among the most important environmental factors in all these diseases [19,20,21]. Obesity, considered as a disease [22] and epidemic in industrialized countries brings obesity as a very critical risk factor for various diseases, [23] including the ongoing COVID-19 pandemic [24,25]. For instance, in 2015, there were over 600 million adults and over 100 million children that were obese worldwide and the numbers are continuously increasing [26]. This epidemic has a tremendous impact on public health, since obesity, especially intra-abdominal fat mass, is associated with a dysregulation of lipoprotein-lipid metabolism and several pathologies and health problems including CVD, T2D, liver disease, impaired regeneration, and cancers [8,27,28,29,30,31,32,33]. Studies have shown that obesity also independently impacts mortality [28,29,34,35]. Weight loss based on either caloric restrictions or pharmacological agents simultaneously improves most CVD risk factors [36,37,38]. However, limited FDA-approved drugs are available, and all of them have important drawbacks. Moreover, body weight management represents a growing and multibillion dollar market [39].

Obesity, also suggested to be a neuroendocrine reprogramming combined to a broken energy homeostasis [40,41], can result from the cumulative effect of a repeated energy imbalance, with minor excesses in energy intake (EI) over energy expenditure (EE) [42]. HF food promotes weight gain through both EI and EE, according to the high caloric density, low satiety effect and high palatability of HF nutrients, as well as the weak potency for fat oxidation and EE that are associated to fat ingestion [19,20,21]. Thus, the acute control of fat intake is a major determinant in the etiology of obesity. Feeding behaviour is controlled by short-term circulating nutrients and hormones, as well as signals derived from the peripheral tissues in response to a meal and changes in energy stores. The hypothalamus is a key brain center upon which all these peripheral signals converge to regulate feeding behaviour and EI [43]. Therefore, as a preliminary step to discover the peripheral factor controlling fat intake and other determinants of energy balance, we previously used functional genomic strategy to investigate gastric, intestinal, fat, and hypothalamic genes differentially regulated by the ingestion of HF and LF meals [44,45,46,47].

Importantly, whereas we have insulin as a signal triggered by glucose that contributes to balancing metabolism and glycemia, there is a need to further explore lipids-specific signals. This would represent an additional step toward controlling/influencing the energy balance with a focus on the HF diet and its related signals.

## 2. Trefoil Factor 2 (*Tff2*) as a High-Fat-Induced Gene

Functional genomics have been proved as a strong tool to characterize many genes specifically induced by different conditions including those related to obesity and within the context of diet and exercise [48,49,50]. For instance, we have identified hundreds of genes modulated after HF or LF meals using the serial analysis of gene expression (SAGE) method [44,45,46,47].

In our previous study [51], the differentially expressed transcripts after LF or HF meals compared to fasting condition were classified into one of the three following patterns: meal responsive (commonly modulated by both meals), LF-specific (modulated only in LF condition) and HF-specific (modulated only in HF condition). Then, using the following criteria, we have selected new candidate genes for controlling appetite and satiety: (1) HF- or LF-specific genes, since lowering fat appetite or increasing satiety could lead to efficient and safe therapies for obesity compared to the interventions decreasing the overall food intake. (2) Genes coding for secreted proteins. Feeding behaviour is controlled by short-term circulating nutrients and hormones as well as signals derived from the peripheral tissues in response to a meal and changes in energy stores. In addition, when the facility of drug delivery method is taken into account, it is reasonable to target secreted proteins. (3) Genes not coding for nutrient’s digestion and absorption. The characterization of proteins involved in lipid digestion and absorption has already been studied for decades. Since the lipase inhibitor Orlistat has undesirable side effects [39], new drugs targeting different pathways or functions are needed. (4) Gene not coding for known appetite/satiety signals, since new candidates are of interest. (5) Genes with potential interest based on published literature. These include genes whose relationship with known appetite/satiety genes has been reported, as well as genes whose involvement in energy balance has been reported but not in feeding behaviour. (6) Patterns and magnitude of expression. These include genes strongly modulated by LF or HF meal, genes showing modulation at interesting time points, and groups of genes showing similar pattern of expressions. (7) Genes with their expression levels confirmed by other methods, such as quantitative real-time PCR (Q_RT-PCR) and Western blot. As a result, trefoil factor 2 (*Tff2*) was the top ranked new candidate gene. Since, we found no work focusing on TFF2 implications within either obesity or HF diet, it was important to focus our next exploration on the mechanisms of TFF2 involvement in energy balance from in vitro to in vivo.

## 3. TFF2 Metabolic Properties and Implications

TFFs constitute a family of polypeptides with a distinctive structural module characterized by six cysteine residues [52]. This structural feature has a stabilizing effect via intramolecular disulfide bridges which are responsible for the remarkable protease resistance of TFFs [53], resulting in a distinct and unusual supersecondary structure clearly identifying the trefoil polypeptide domain as a unique growth factor-associated module, structurally unrelated to other highly disulfide-linked modules such as those in epidermal growth factor and insulin-like growth factor-I [54,55,56,57]. TFFs are small secreted proteins involved in protection of the mucosal lining of the gastrointestinal tract [58,59]. They protect against gastrointestinal damage by stimulating the migration of adjacent epithelial cells, a process termed “restitution”, and by virtue of their interaction with mucins and other proteins [60,61,62].

Tissue localization analyses show the highest expression of TFF2 (also known as SP; SML1) in stomach/duodenum [63]. TFF2 has structural homology with growth hormone and has been detected in blood streams [54,64,65]. Moreover, *Tff2* is specifically upregulated by HF meals in gastrointestinal mucosa, whereas downregulated in hypothalamus [44,66]. This is in agreement with the expression pattern of a peripheral signal secreted to inform the brain to stop eating fat. We have shown that *Tff2* knockout (KO) mice have increased EI, EE, and excretion, as well as lower abdominal adipose tissue [66].

Studies with *Tff2*-deficient mice have not only shown the evidence of decreased gastric cell proliferation, increased acid secretion, and increased susceptibility to gastric mucosal injury [67], but have also revealed new aspects of *Tff2*’s role in the immune response such as changes in the expression of diverse crucial genes involved in innate and adaptive immunity [68,69], including defensins which regulate the composition of the intestinal bacterial microbiome [70]. The innate and adaptive immunity as well as microbiota are dysregulated in obesity and metabolic disorders such as insulin resistance [71,72]. Moreover, TFF2 secretion can be regulated by both pro-inflammatory and anti-inflammatory cytokines including tumor necrosis factor alpha (TNF-α), interleukin 4 (IL-4), and IL-13, and in turn influence cytokine release and activation (i.e., IL-1, IL-6) [69], as well as immune cell recruitment [73]. This correlates with its suggested roles both as anti-inflammatory [74] and in reducing immune-mediated damage [75].

Furthermore, expression of apolipoprotein A-IV *(Apoa4*) which has an anti-inflammatory effect and inhibits gastric motility, emptying and acid secretion [76,77], has been shown to be upregulated in the stomach and duodenum of *Tff2*-deficient mice [68,69]. Importantly, apoA-IV is a satiety signal induced by lipid ingestion [78]. Moreover, the circadian rhythm of pancreatic polypeptide expression, which is known to regulate food intake, is negatively correlated with gastric *Tff2* circadian rhythm [79]. *Tff2* expression has been shown to be regulated by peroxisome proliferator-activated receptor (PPAR) γ [80], which is established as an important target for the treatment of T2D and other disorders associated with HF intake [81]. Finally, the repression of *Apoa4* was followed by *Tff2* whose modulation was concomitant with fat intake [44]. Therefore, this evidence suggest that *Tff2* might be a novel candidate gene for fat satiety control. However, the roles of this protein in the regulation of feeding behaviour, energy balance and development of obesity are yet to be fully understood. Therefore, the characterization of the mechanisms will be the first step to understand its roles, as well as to develop new therapeutic targets to control food and fat intakes.

It is widely accepted that TFFs exert their biological action through a cell surface receptor [82]. Concordantly, several membrane proteins are found to interact with TFF2 [83,84,85,86]. Recently, Dubeykovskaya et al. demonstrated an ability of TFF2 peptide to activate Ca^2+^-Akt-ERK1/2 (for extracellular-signal-regulated kinases 1/2) signaling pathway via chemokine (C-X-C motif) receptor 4 (CXCR4). Interestingly, CXCR4 was first reported as bovine neuropeptide Y (NPY) Y3 receptor [87]. Moreover, following 12 weeks of feeding, *Tff2* KO mice exhibited lower abdominal adipose tissue and diameter of retroperitoneal adipocytes compared to the wild-type mice [66]. Furthermore, the *Tff2* KO mice showed increased EE and excretion, whereas higher EI were observed. Digestive energy efficiency, duodenum mucosal length, glycemia, as well as insulin and leptin levels, were lower in KO mice [66]. Appetite signal, agouti-related protein (*Agrp*), expression was higher in the hypothalamus [66]. Thus, TFF2 seems to be a mastermind regulator of overall energy balance. Consequently, it is important to investigate the various mechanisms of action of TFF2 on energy balance corresponding not only to new, but also multiple opportunities to eventually prevent and treat obesity, which remains to this day a major challenge to public health providers.

In order to identify the mechanisms whereby TFF2 regulates feeding, we need to explore the pathways of TFF2 action on feeding. *Tff2* KO mice showed HF-specific increase in meal intake [66]. Any satiety signal induced by food intake (FI) may reach the central nervous system (CNS) either through blood circulation or vagal nerve afferences relaying gastrointestinal stimuli. Since *Tff2* is expressed in the gastric mucosa, and that oral/systemic administration of radioactive TFF2 was found in blood, digestive tracts, and brain, TFF2 might act as a peripheral signal to the CNS [64]. It is worth noting that no adverse metabolic effect has been reported with oral/systemic administration of TFF2 [64].

**Figure 1:**
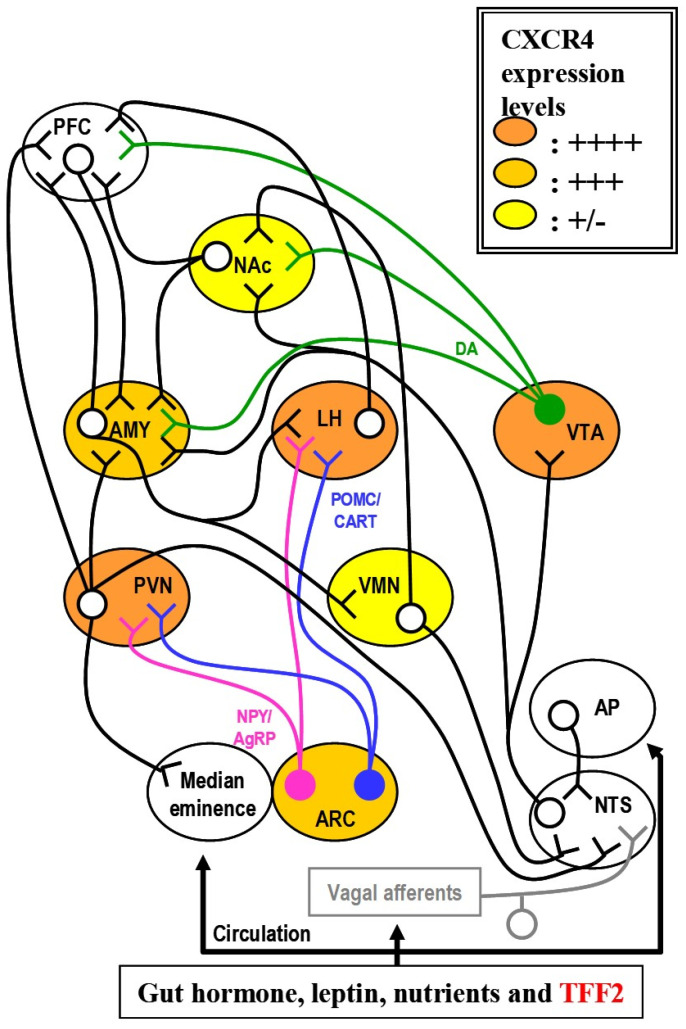
Possible connection between CXCR4 and appetite/satiety signals. Meal-modulated hormonal and neuronal signals from the gut are received via the blood in the area postrema (AP) and through vagal afferent fibers in the nucleus of the solitary tract (NTS), respectively. These sensory inputs are transmitted via the ventral tegmental area (VTA) to other centers, including the amygdala (AMY) and nucleus accumbens (Nac), where dopaminergic (DA) and other signals act in reward processes. Inputs from these pathways are integrated with circulating signals of nutritional state which are detected in the arcuate nucleus (ARC) via the median eminence. Within the ARC, the activity of neurons expressing proopiomelanocortin (POMC) is stimulated, while that of neurons expressing neuropeptide Y (NPY) is inhibited by leptin. Axons from both types of neurons project in parallel to the paraventricular nuclei (PVN) and lateral hypothalamus (LH). Release of α-melanocyte-stimulating hormone by POMC-expressing neurons leads to activation of the melanocortin receptor 4 (MC4R), which lowers food intake and increases energy expenditure (EE). By contrast, release of NPY activates Y1 and Y5 receptors, which increases food intake and reduces EE. NPY-expressing neurons also release agouti-related protein (AgRP), an antagonist of MC4R. This dual innervation within the PVN modulates EE via the thyroid and adrenal axis and the sympathetic nervous system. Abbreviations: CART, cocaine–amphetamine regulated transcript; CXCR4, chemokine (C-X-C motif) receptor 4; PFC, prefrontal cortex; TFF2, trefoil factor family member 2; VMN, ventromedial nuclei.

On the other hand, regarding the biological links, there are connections between CXCR4 and appetite/satiety signals (possibly involving TFF2, Figure 1). Indeed, TFF2 receptor, CXCR4, is localized within areas rich in dopaminergic (DA) neurons [88]. Mesolimbic/mesocortical DA neurons are essential for reward and motivational behaviours [89]. Thus, there would be an important addition to elucidate whether CXCR4 is expressed in the same neurons expressing appetite (*Npy*/*Agrp*)/satiety (proopiomelanocortin, *Pomc*/cocaine-amphetamine regulated transcript, *Cart* and corticotrophin releasing hormone, *Crh*) signals. We have shown higher *Agrp* expression in the hypothalamus of *Tff2* KO mice [66]. To elucidate how TFF2 modulates energy expenditure, there is a need to focus on energy expenditure since there are evidences that a regulatory control on EE is exerted through the sympathetic nervous system, especially on BAT thermogenesis by uncoupling protein 1 (UCP1) [90]. The TFF2 receptor, CXCR4 is involved in mesolimbic/mesocortical DA system to modulate locomotor activity and our data showed higher locomotion activity in *Tff2* KO mice [91]. These suggest the involvement of mesolimbic/mesocortical DA system in the modulation of locomotion activity in *Tff2* KO mice via CXCR4. Therefore, behavioural activity after TFF2 injection with or without pre-infusion of CXCR4 is to be investigated as well [66,91]. Importantly, expression of CXCR4 in other tissues including digestive tract and metabolic tissues [92,93,94,95,96,97,98,99,100], could suggest a metabolic action of TFF2 via CXCR4 within these tissues.

Regarding the EE during HF or LF feeding, we have demonstrated that *Tff2* KO mice exhibited higher locomotor activity and total EE than WT animals [66]. The metabolic exploration of *Tff2* KO mice revealed interesting patterns for the lipid intake and excretion as well as the energy balance within the key metabolic tissues, as well the body and tissue weights [101]. Briefly, the *Tff2* KO in mice resulted in lower glucose, triglycerides (TG), and glycerol serum levels with a metabolism towards less fat storage and increased EE by enhancing lipid and glucose utilization via oxidative phosphorylation in the key metabolic tissues (muscle, liver and adipose tissues) [101]. The *Tff2* KO also led to reduced body and adipose tissues weights [101].

## 4. Experimental Perspectives

In order to complete the puzzle surrounding the molecular mechanisms and pathways of the multifunctional TFF2 in the context of lipid-related signals along with a possible connection between fat sensing in gut and the satiety signals in hypothalamus, further studies can be suggested.

Following fat digestion by lipases, the lipolytic products are absorbed by the enterocytes where chylomicrons are formed and secreted. The assembly of these TG-rich lipoproteins within the enterocyte is a multistep pathway including: (1) the uptake (CD36 and FA transport protein 4, FATP4) and translocation of lipolytic products from the brush-border membrane to the endoplasmic reticulum by FA binding protein 1/2 (FABP1/2); (2) TG synthesis via monoacylglycerol pathway (monoacylglycerol acyltransferase 2, MGAT2 and diglyceride acyltransferase 2, DGAT2) and phosphatidic acid pathway; (3) the packaging of lipid and apolipoprotein components into lipoprotein particles (apoB-48 and apoA-IV); (4) the transport into the Golgi for secretion (microsomal TG transport protein, MTP) [102]. We have already reported that many genes listed above have been modulated by HF and LF meal ingestion [44]. Thus, the expression levels of such key genes and proteins involved in lipid absorption are important to understand.

There are several practical steps that would also allow further exploration of TFF2 metabolic implications. These include injection of this secreted (recombinant protein) protein to mice orally, intravenously and intracerebroventricularly [103,104,105,106,107,108,109,110,111,112,113]. Brain sections can be processed for immunocytochemical detection of CXCR4 [88], and in situ hybridization of *Npy*, *Agrp*, *Pomc*, *Cart* and *Crh* in mice either fasted or refeeding with HF or LF meal to make a link between the expression of the molecules and the corresponding *Tff2* expression depending on the status (fasting, LF feeding and HF feeding). Along with the expression of other peripheral appetite/satiety signals (ghrelin, cholecystokinin, PYY, and apoA-IV) [114] combined with c-Fos expression that is particularly reliable to assess brain activations in response to feeding [103], an in situ hybridization of *Npy*, *Agrp*, *Pomc*, *Cart* and *Crh* mRNA can be performed to determine whether NPY/AgRP, POMC/CART and CRH cells will be activated [103,105,106]. It has been shown that c-Fos expression is induced by refeeding [103], and the subcutaneous injection of peptide YY (PYY) in the nucleus of the solitary tract (NTS) [115] which represents an additional molecular link. In addition, an in vitro model such as Caco-2 cells, used as a valid model of study for lipid/lipoprotein homeostasis [114,116], would allow us to study the lipid synthesis, secretion, and metabolism variations with both *Tff2* knockdown and overexpression. Similar studies can be conducted in the diverse existent animal models of obesity as well [117].

## 5. Conclusions

**Figure 2:**
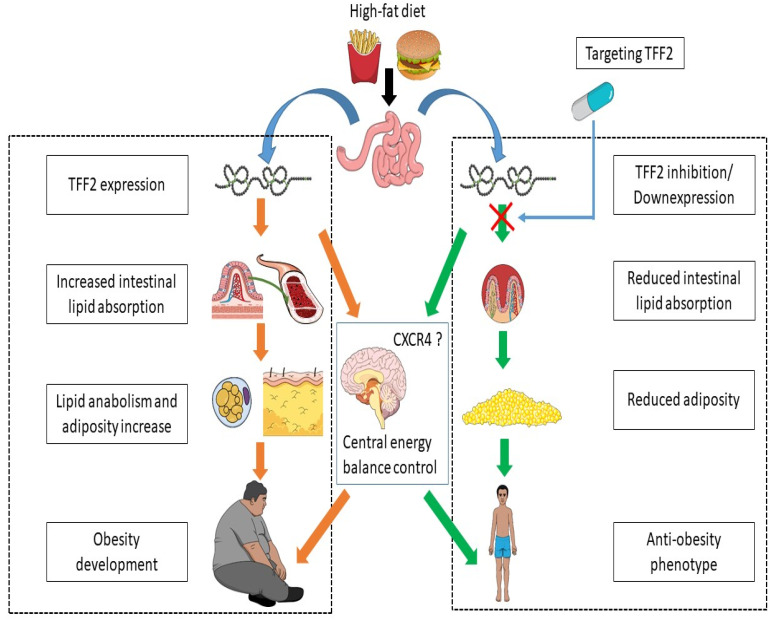
TFF2 hypothetic implications in obesity development and its potential therapeutic targeting. The high-fat diet increases TFF2 expression. This would lead to the facilitation of the intestinal lipid absorption, as well as the increase in lipids storage in part, at least, via increasing anabolism. All these contribute to obesity development. Thus, inhibiting or reducing the expression or the action of TFF2 would reduce the lipid intestinal absorption as well as the adiposity. That points targeting TFF2 and its related pathways as a potential antiobesity therapeutic approach. Both TFF2 action and inhibiting TFF2 mechanisms would involve CXCR4 pathways. Abbreviations: CXCR4, chemokine (C-X-C motif) receptor 4; TFF2, trefoil factor family member 2.

The functional genomics studies that revealed *Tff2* as a HF-induced gene combined with the observations of the metabolic implications of TFF2, as well as the metabolic phenotype of *Tff2* KO mice allow us to build a hypothetic path regarding a TFF2 mechanism as a response to HF diet (Figure 2). The HF diet induces an overexpression of TFF2 which acts not only towards energy balance centers, (Figure 1) but would also facilitate the lipids intestinal absorption. This explains that the *Tff2* KO mice had an increased energy excretion in form of TG (lipids not absorbed). In addition, the *Tff2* KO resulting in an increased metabolic activity of both lipids and glucose in the mice (that ultimately led to the protection from the HF diet-induced obesity) points TFF2 as a lipid anabolic (and possibly a carbohydrates conversion into lipids) factor and explains the reduced adiposity in *Tff2* KO mice. On the other hand, the other properties associated to TFF2 such as anti-inflammatory [74] and reducing immune-mediated damage [75] would indicate roles that would balance some of the HF diet consequences, such as inflammation and immune induction. This correlates with the protective roles TFF2 plays in the digestive mucosa as well. Since during fat absorption apoA-IV is secreted into intestinal lymph [118], the upregulation of Apoa4 in *Tff2*-deficient mice [68,69] could be a regulatory mechanism aiming to compensate the reduced lipid absorption resulting from the absence of TFF2. Taken together, these elements highlight TFF2 as a molecule induced by HF diet in order to facilitate the absorption of the lipids, their storage, as well as their anabolism, along with the induction of mechanism that would correct or reduce the negative impacts of HF diet in terms of inflammation and immune impacts. Ultimately, we would conclude that TFF2, as a lipid anabolic factor and a lipid absorption facilitator, would contribute to obesity establishment. Such important molecular implication in obesity pathogenesis fits well and explains the reported protection from HF diet-induced obesity seen in *Tff2* KO mice.

Within this context, there is a potential to develop new treatments for obesity and related diseases by characterizing the molecular mechanisms by which TFF2 controls energy balance and target the related pathways. The next objectives are to identify the mechanisms by which TFF2 regulates feeding, EE, and energy excretion, as well as explore the roles of TFF2 in obesity and related diseases. Future studies will allow the characterization of potential therapeutic targets which can be used for the treatment of obesity and related diseases by the administration of the pharmaceutical inhibitors of TFF2 pathway among other options. The ultimate goal is to develop a novel generation of treatments and therapeutic targets for obesity and metabolic disorders that would pharmacologically target TFF2 pathways to mimic the antiobesity effects of *Tff2* KO (Figure 2). The putative advantages of interference with intestinal lipid absorption in course of TFF2 blockade compared to the well-established therapeutic option of lipase inhibition by orlistat could be summarized within several points. Unlike orlistat [119,120], the *Tff2* KO mice did not have diarrhea, although they had an increased TG excretion [66]. TFF2 blockade would also lead to an increased EE. In addition, targeting TFF2 pathway would lead to an antiobesity effect not only via the interference with intestinal lipid absorption but also through the energy metabolism control center as well as the key metabolic tissues. However, the involvement of TFF2 in different functions [86,121,122,123], such as mucosa protection, imposes a specific pharmacovigilance for such potential pharmacological approaches such as prioritizing targeting the peripheral (intestinal) TFF2 pathways rather than the central (brain) pathways, such as using a CXCR4 antagonist. Importantly, since intestinal villi increase the absorptive area and the surface area of the intestinal wall and the implication of TFF2 in the mucosa, possible morphological changes of such pharmacological targeting could affect nutrients absorption as well. In addition, an assessment of metabolic markers in the key metabolic tissues including those revealed by the metabolic exploration of *Tff2* KO mice [101] would be important for such pharmacological agents. That includes the fatty acids translocase in the skeletal muscle, as well as the nerve growth factor IB (NGFIB, also known as Nur77) that both coordinately regulates the expression of genes linked to glucose metabolism, including insulin sensitive glucose transporter type 4 (*Glut4*) [124] and regulates lipolysis and gene expression of *Ucp2/3*, *Pgc1α*, *Cd36* [125,126]. In the liver, Nur77 stimulates glucose production and induces expression of genes involved in gluconeogenesis [126]. Adiponectin, which is exclusively secreted from adipose tissue, also affects these insulin-sensitizing processes by stimulating fatty acids oxidation in the skeletal muscle and repressing hepatic-glucose production [127]. PPARγ is a master regulator of adipocyte differentiation and function [128]. Thus, along with leptin and adiponectin, these elements are at the heart of the molecular explorations required towards developing “lipid metabolism-controlling pills”.
